# Primary Vaginal Myeloid Sarcoma: A Rare Case Report and Review of the Literature

**DOI:** 10.1155/2015/957490

**Published:** 2015-01-18

**Authors:** Gaurang Modi, Irappa Madabhavi, Harsha Panchal, Apurva Patel, Asha Anand, Sonia Parikh, Pritam Jain, Swaroop Revannasiddaiah, Malay Sarkar

**Affiliations:** ^1^Department of Medical and Paediatric Oncology, GCRI, Ahmedabad, Gujarat, India; ^2^Department of Radiotherapy, Government Medical College, Haldwani, India; ^3^Department of Pulmonary Medicine, IGMC, Shimla, Himachal Pradesh, India

## Abstract

Myeloid sarcoma (chloroma, granulocytic sarcoma, or extramedullary myeloid tumour) is an extramedullary mass forming neoplasm composed of myeloid precursor cells. It is usually associated with myeloproliferative disorders but very rarely may precede the onset of leukemia. Here, we are presenting a rare case of primary vaginal myeloid sarcoma in a geriatric female patient without initial presentation of acute myeloid leukemia (AML). A 68-year-old female patient with ECOG Performance Score of 1 presented with pervaginal bleeding for 20 days. On colposcopic examination, she was found to have mass in the anterior fornix of vagina. A punch biopsy specimen revealed chloromatous infiltration of the vagina. LCA (leukocyte common antigen), MPO (myeloperoxidase), and c-kit were strongly positive on IHC (immunohistochemistry). The patient's routine blood investigations were normal including peripheral smear, lactose dehydrogenase, uric acid, 2D echocardiography, conventional cytogenetics, bone marrow aspiration, and biopsy. The patient was given 4 cycles of decitabine (Decitex, manufactured by Sun Pharmaceutical Industries Limited, India), 20 mg/m^2^ for 5 days at an interval of 28 days. There was a partial response to decitabine according to RECIST criteria. As decitabine therapy was well tolerated, we are continuing in the same way until disease progression without any complications. The patient is undergoing regular follow-up at our centre.

## 1. Introduction

Myeloid sarcoma (chloroma, granulocytic sarcoma, or extramedullary myeloid tumour) is an extramedullary tumour composed of immature myeloid cells with varying degree of maturation. Isolated myeloid sarcoma, defined by the absence of history of leukemia, myelodysplastic syndrome (MDS) or myeloproliferative neoplasm, and a negative bone morrow biopsy, has been described in limited case reports. Myeloid sarcoma is a rare manifestation of leukaemia and has been reported in 3–5% of acute myelogenous leukemia (AML) patients [1].

## 2. Case Summary

A 68-year-old female with ECOG (Eastern Cooperative Oncology Group) Performance Score of 1 presented with complains of irregular vaginal bleeding for 20 days. There was no history of weight loss, fever, or night sweats, and the blood pressure was 136/84 mmHg. On examination, her height, body weight, and body mass index were within normal range for her age.

**Figure 1:**
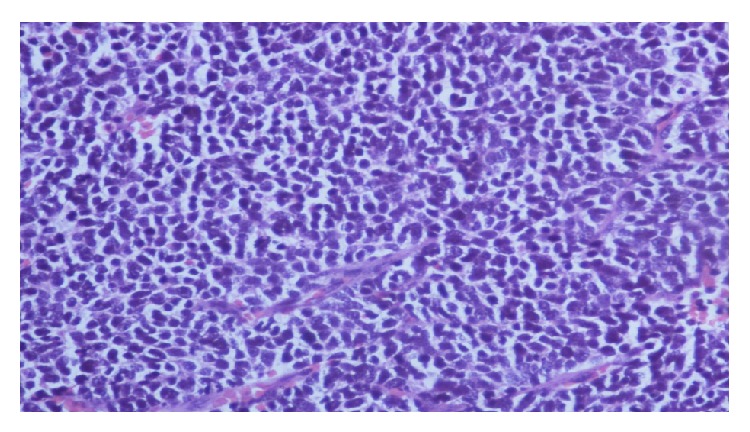
Histopathological image showing diffuse infiltration of the vagina by mononuclear cells which has prominent nucleoli with scanty cytoplasm.

**Figure 2:**
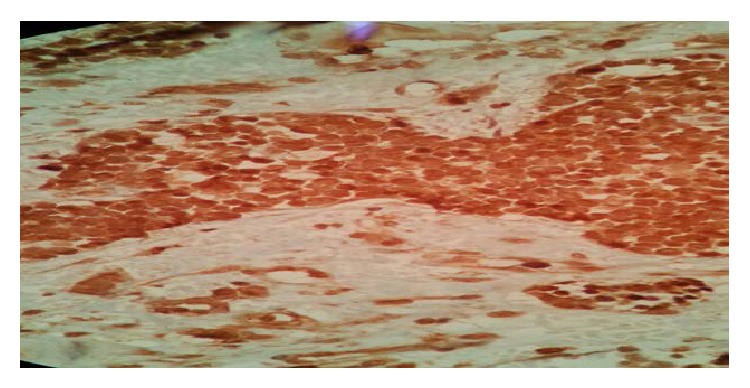
IHC image showing positivity for myeloperoxidase (MPO).

**Figure 3:**
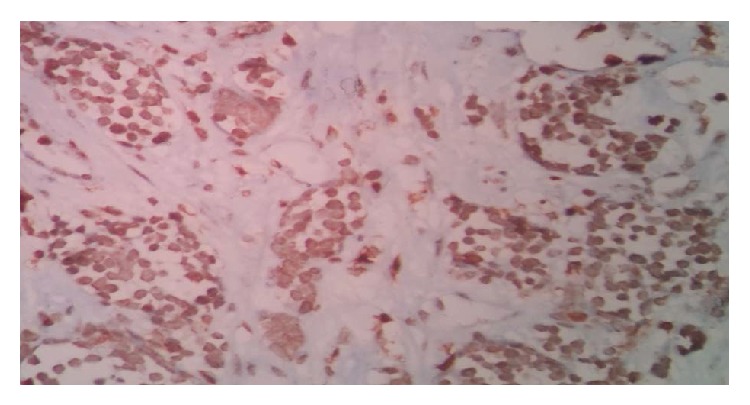
IHC image showing positivity for leukocyte common antigen (LCA).

**Figure 4:**
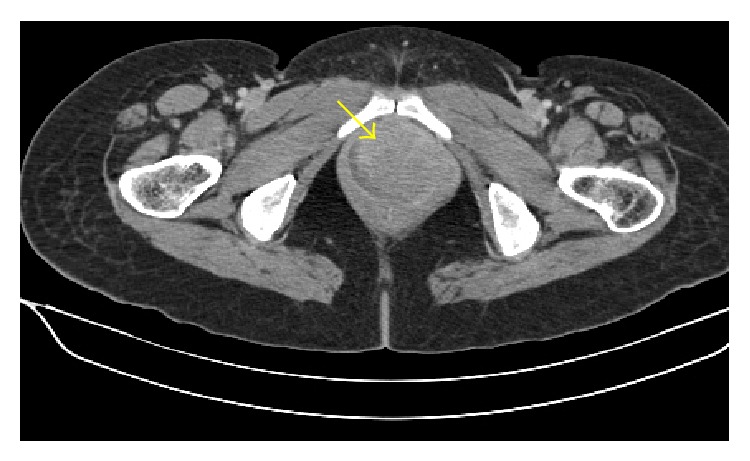
Computed tomography (CT) image of the pelvis shows well-defined homogenously enhancing lesion which is arising from left sided anterolateral wall of the vagina and projecting into vaginal lumen.

On colposcopic examination, she was found to have a 5 × 6 cm polypoidal mass in the anterior fornix and left lateral wall of vagina. The rest of the systemic examination was unremarkable. Histopathological examination of the punch biopsy specimen from the mass revealed diffuse infiltration of the vagina by mononuclear cells which had prominent nucleoli with scanty cytoplasm (Figure 1). The biopsy specimen showed positivity for MPO (myeloperoxidase) (Figure 2), leukocyte common antigen (LCA) (Figure 3), and c-kit on IHC (immunohistochemistry). The biopsy specimen was negative for cytokeratin, synaptophysin, chromogranin, CD20, and CD99 on IHC. The patient's complete hemogram and peripheral smear, renal function tests, liver function tests, blood sugar, lactose dehydrogenase, and uric acid were within normal limits. The patient's bone marrow aspiration, trephine biopsy, and conventional cytogenetic were also normal. Computed tomography (CT) image of the pelvis showed a well-defined homogenously enhancing lesion arising from the left sided anterolateral wall of the vagina and projecting into vaginal lumen (Figure 4).

A diagnosis of primary (isolated) vaginal sarcoma was made from the above reports. Considering her age and comorbidity, we offered her hypomethylating therapy with decitabine (Decitex, manufactured by Sun Pharmaceutical Industries Limited, India). Decitabine was given intravenously at the dose of 20 mg/m^2^ for 5 days at an interval of 28 days for 4 cycles. There was a partial response to decitabine according to RECIST criteria in CT scan done after 4 cycles. As there were no tolerance issues, we are continuing with decitabine until disease progression without any complications.

## 3. Discussion

Acute myeloid leukemia may present in a variety of extramedullary (EM) tissues with or without bone marrow disease. Extramedullary involvement by AML is relatively rare, but clinically often poses diagnostic challenge and therapeutic dilemma. It was first described in 1811 and later named “chloroma” by King in 1853 because of its green colour caused by the presence of myeloperoxidase (MPO) [2]. Although there have been various synonyms for an extramedullary myeloid tumour, myeloid sarcoma is currently recommended by world health organization.

Very rarely, myeloid sarcoma can occur without a known preexisting or concomitant diagnosis of acute leukemia, acute promyelocytic leukemia, or MDS/myeloproliferative syndrome (MPS); this is known as primary myeloid sarcoma. In almost all reported cases of primary myeloid sarcoma, acute leukemia had developed shortly afterward (median time to development of acute leukemia: 7 months, range: 1–25 months) [3]. Therefore, primary myeloid sarcoma could be considered an initial manifestation of acute leukemia, rather than a localized process, and could be treated as such.

Myeloid sarcoma is reported in 2.5–9.1% of patients with AML and occurs concomitantly, following, or, rarely, antedating, the onset of systemic bone marrow leukemia [4]. Certain known AML cytogenetic abnormalities, like t(8; 21), have been associated with a higher incidence. In our case, there was not any translocation. Myeloid sarcoma can also develop at relapse with or without marrow involvement. The frequency with which certain myeloid sarcoma sites are accompanied by marrow involvement has not been adequately studied. Clinical manifestations are varied which is dependent on the various sites and sizes at presentation. The most common locations include the soft tissue, bone, periosteum, and lymph nodes. Clinically, significant involvement of the female genital tract is rare. The most commonly involved organ is the ovary estimated at 36.4% followed by the cervix and uterus but vaginal involvement is very unusual as in our case [5]. Most of the patients (81–83%) present with vaginal bleeding similar to our case [6].

The diagnosis is not always easy when chloroma appears at an EM site especially when AML is not present. Most of them are poorly differentiated, and only in 44% of cases the correct diagnosis is made or suspected. The most common misdiagnosis is the high grade non-Hodgkin lymphoma because both the conditions are composed of diffusely infiltrating, discohesive cells that tend to spare normal structures and which may contain scattered lymphocytes. In myeloid sarcoma, however, the nuclei are typically slightly smaller with more finely dispersed chromatin, and some cell may show recognizable myeloid differentiation. The immunohistochemical stains are usually diagnostic [5].

The optimal treatment of isolated myeloid sarcoma (MS) is unclear, given the rarity of the diagnosis, variability in presentation, and the lack of prospective studies. Treatment is variable and often delayed. Treatment of isolated MS similar to leukemic AML and induction chemotherapy is now the standard of care. It is notable that isolated MS almost always proceeds to frank leukemia, although cases without progression even upon long-term follow-up have been reported [7]. Currently, surgical and radiation therapy are accepted treatment modalities; however, their precise roles in the treatment algorithm are not well defined. Rapid symptomatic relief, initial debulking, inadequate response to chemotherapy, and recurrence after hematopoietic stem cell transplantation (HSCT) are some of the indications for these ancillary therapeutic modalities. Thus, treatment options are local radiotherapy (RT) and systemic chemotherapy [7]. Local RT can be used to hasten control of symptoms when vital organ is involved. Role of postremission radiotherapy is controversial. Few reports of complete remission have been described after aggressive multimodality treatment of myeloid sarcoma in other sites including disseminated myeloid sarcomas without evidence of AML.

Lan et al. studied 24 patients of myeloid sarcomas and found that 5-year survival rate was approximately 20%. In this study, patients undergoing chemotherapy had a significantly longer survival time compared to those who did not (*P* = 0.0009) and we found no difference in the 5-year survival rate among the patients undergoing chemotherapy combined with radiation or surgery [8]. Because of the toxicity and uncertain benefit of standard induction chemotherapy in the older population of patients with AML, many of these patients are offered only supportive care or low intensity chemotherapy [9, 10]. Considering the age of the patient (68 years) and comorbid conditions like hypertension, it was assumed that she would not tolerate conventional 7 + 3 induction (3 days of daunorubicin and 7 days of cytarabine). Data suggests that aggressive induction chemotherapy in elderly patients is poorly tolerated and results in a higher rate of mortality. Thus, treatment was even more challenging in this elderly patient with myeloid sarcoma.

Recently, there has been considerable interest in the hypomethylating agents like decitabine and 5-azacitidine as alternative treatment option for elderly patients with AML. Decitabine is hypomethylating agent which selectively inhibits DNA methyltransferases and it is a cell cycle specific (S phase) drug. Incorporation of decitabine triphosphate into DNA results in inhibition of DNA methyltransferases (DNMT). This reduces the DNMT activity in cells and leads to hypomethylation of DNA, resulting in the reexpression of genes necessary for the control of cellular differentiation and proliferation [11].

Decitabine has been approved by the European Medicines Agency (EMA) for the treatment of adult patients aged ≥65 years with newly diagnosed de novo or secondary AML, according to the WHO classification, who are not candidates for standard induction chemotherapy. Decitabine should be administered by intravenous infusion over 1 h at a dose of 20 mg/m^2^ body surface area each day for the first 5 consecutive days of a 4-week cycle. It is recommended that patients receive at least 4 cycles; however, it may take longer than 4 cycles to obtain a complete or partial remission. Treatment may be continued as long as the patient shows response, continues to benefit, or exhibits stable disease. It is generally well tolerated, with most adverse events being related to myelosuppression [11]. Phases 2 and 3 studies have demonstrated that decitabine, at a variety of doses and schedules, has activity in AML [12–15]. Kantarjian et al. studied decitabine in phase III trial comparing it with patient choice, with physician advice, of supportive care (SC) or cytarabine in older patients with AML. Study concluded that decitabine improved response rate compared with standard therapies without major differences in safety. This updated analysis of the trial data (with an additional year of follow-up) demonstrated the same 2.7-month improvement in median overall survival with decitabine versus TC, but, in this analysis, the difference between groups was significant (nominal *P* = 0.037). This significant survival benefit was not observed at the time of the primary analysis [14].

There are few published case reports validating efficacy of hypomethylating agents in extramedullary AML [16, 17]. Singh et al. have described durable complete remission after single agent decitabine in AML relapsing in extramedullary sites after allo-SCT. The patient had complete remission with normal counts and had received a total of 13 cycles of decitabine therapy with no serious side effects [16].

Isolated myeloid sarcoma patients require long term follow-up as extramedullary recurrences are frequent. Recurrences may be at the same or distant sites [18]. Relapsed or refractory disease is observed with or without bone marrow involvement. There is very limited data about managing such relapsed patients. Our patient has been advised of regular follow-up at our centre.

## 4. Conclusion

Myeloid sarcoma involving vagina in a geriatric patient without AML is a very unusual presentation and it is a diagnostic and therapeutic challenge. Clinical suspicion is the key to correct diagnosis and expert pathology opinion and immunochemistry tests are required to confirm it. Treatment options include systemic treatment for AML, with or without local therapies. It is important to consider individual case differences, for example, age (elderly) as in our case. Our case illustrates the fact that isolated vaginal chloroma can be treated palliatively with hypomethylating agent decitabine in an appropriate setting.
